# Changes in anaesthetic use for trauma patients in German HEMS – a retrospective study over a ten-year period

**DOI:** 10.1186/s13049-019-0603-9

**Published:** 2019-02-26

**Authors:** Arasch Wafaisade, Michael Caspers, Bertil Bouillon, Matthias Helm, Matthias Ruppert, Michael Gäßler

**Affiliations:** 1 Department of Traumatology and Orthopaedic Surgery, Cologne-Merheim Medical Centre (CMMC), Witten/Herdecke University, Campus Cologne-Merheim, Ostmerheimer Str. 200, 51109 Cologne, Germany; 2 The Institute for Research in Operative Medicine, Faculty of Health, Department of Medicine, Witten/Herdecke University, Ostmerheimer Str. 200, 51109 Cologne, Germany; 3 Armed Forces Medical Centre Ulm, Department of Anaesthesiology and Intensive Care Medicine, Section Emergency Medicine – HEMS “Christoph 22”, Oberer Eselsberg 40, 89081 Ulm, Germany; 4 Department of Medicine – ADAC Air Rescue Service, Hansastrasse 19, 80686 Munich, Germany

**Keywords:** Anaesthetic, Trauma, Sedative, Opioid, Pre-hospital, Trauma care, HEMS

## Abstract

**Background:**

Airway management and use of intravenous anaesthetics to facilitate tracheal intubation after major trauma remains controversial. Numerous agents are available and used for pre-hospital rapid-sequence induction (RSI). The aim was to investigate usage and potential changes in administration of intravenous anaesthetics for pre-hospital RSI in trauma patients over a ten-year period.

**Methods:**

Based on a large helicopter emergency medical service (HEMS) database in Germany between 2006 and 2015, a total of 9720 HEMS missions after major trauma leading to RSI on scene were analysed. Administration practice of sedatives and opioids were investigated, while neuromuscular blocking agents were not documented in the database.

**Results:**

With respect to administration of sedatives, independent from trauma mechanism and specific injury patterns the use of Etomidate decreased dramatically (52 to 6%) in favour of a more frequent use of Propofol (3 to 32%) and Ketamine (9 to 24%; all *p* < 0.001) from 2006 to 2015. The use of Benzodiazepines increased slightly, while the utilization rate of Barbiturates remained constant. In patients with Shock Index > 1 at initial contact, the administration rate of Etomidate dropped significantly as well. This decline was mainly substituted by Ketamine and particularly Propofol. In patients with GCS ≤ 8 upon initial contact, a similar distribution compared to the general trauma population could be observed.

With respect to opioids, mainly Fentanyl has been administered for RSI in trauma patients (2006: 69,6% to 2015: 60.2%; *p* < 0.001), while the use of sufentanyl showed a significant increase (0.2 to 8.8%; *p* < 0.001).

**Conclusions:**

This large study analysed prehospital administration of anaesthetics in trauma patients, showing a substantial change from 2006 to 2015 despite the lack of any high-level evidence. Etomidate has shifted from the main sedative substance to virtual absence, indicating that the recommendation of an established national guideline was transferred into clinical practice, although based on weak evidence as well. The pre-hospital use of Propofol showed a particular increase. Fentanyl has been the main opioid drug for RSI in trauma, however Sufentanyl has become increasingly popular. The mechanisms and advantages of the different substances still have to be elucidated, especially in head injury and bleeding trauma.

## Background

Despite a high degree of standardization in pre-hospital trauma care, airway management and more particularly the use of intravenous anaesthetic drugs to facilitate tracheal intubation remains controversial. However, beyond dispute the first priority in trauma management is the evaluation and affirmation of a patent airway and to ensure adequate oxygenation and ventilation [1]. Thereby, poor airway management both pre-hospital and upon hospital arrival continues to be identified as an avoidable cause of morbidity and mortality [2]. A farsighted and secure airway management is of particular importance. Delay in adequate management in patients that are initially stable and presenting rapid aggravation may have devastating consequences going along with increased mortality [3–5]. Thereby, the decision on the respective intravenous anaesthetic for rapid sequence induction (RSI) plays a decisive role.

However, currently numerous sedative agents are available for pre-hospital RSI including Etomidate, Propofol, Barbiturates (Thiopental), Phencyclidines (Ketamine) and Benzodiazepines (Midazolam) [6]. Pharmacokinetics and –dynamics of all these agents are well-known, but due to the particular vulnerable situation in trauma care, attenuation of drug-specific side-effects, like cardiovascular depression, is a major concern during RSI on scene.

During the last years several guidelines of different professional societies participating in trauma management were published [1, 6–9]. Still, there is no consensus and a wide variety of on-scene anaesthetics administration depending on individual patient status, preferences of the treating emergency physician and local policies.

Based on a large HEMS (helicopter emergency medical service) database the aim of the current study was to investigate usage and potential changes in administration of different intravenous anaesthetics (sedatives and opioids) for RSI on scene after trauma over a ten-year period.

## Methods

### Study design

The present study is based on data from a Helicopter Emergency Medical Service. The ADAC Air Rescue Service operates 35 air rescue bases throughout Germany and is therefore one of the largest air rescue providers in Europe. The medical crews of all bases consist of an experienced emergency physician and a paramedic (HEMS Technical Crew Member). In 2015, more than 54.000 rescue missions were performed. The medical authorities of the ADAC Air Rescue Service approved design and publication of this study.

**Fig. 1:**
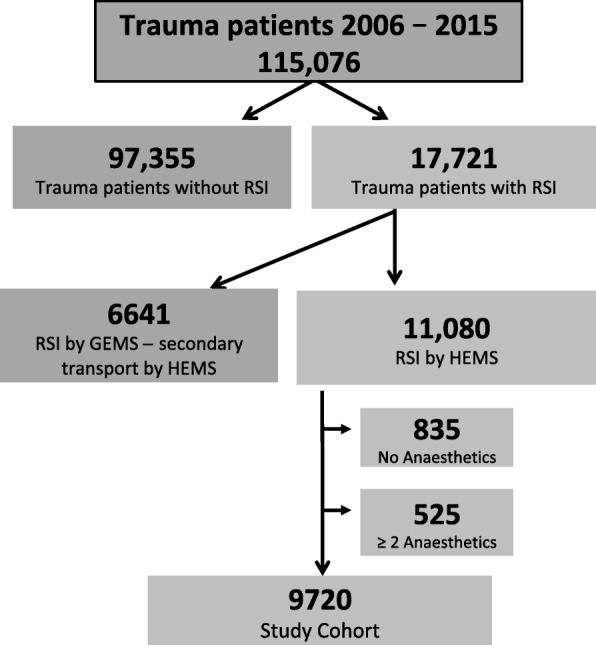
Cohort description of all trauma patients documented within the LIKS database between 2006 and 2015. (GEMS = ground emergency medical service; HEMS = helicopter emergency medical service; RSI = rapid sequence induction)

As shown in the study outline in Fig. 1, between January 1st 2006 and December 31st 2015, a total of 115,076 trauma patients were included in the database. Then, trauma patients that did not receive RSI (*n* = 97,355) were excluded. Also, patients who were intubated prior to HEMS arrival were not eligible for this study. Cases were also excluded if no anaesthetic drug was documented or if more than one intravenous sedative was used for induction. The listed injury pattern and severity have been documented in the database according to the on-scene clinical assessment of the treating emergency physician.

In general, prehospital anaesthesia is conducted as rapid-sequence induction (RSI). In the following the term “anaesthetic” comprises both sedatives (Propofol, Etomidate, Thiopental, Ketamin/Ketamin S, Midazolam) and opioids (Fentanyl, Sufentanyl, Morphine, “Mix”), that were both analysed, with subgroup analyses for the sedatives. The use of neuromuscular blocking agents (NMBA) was not analyzed in this study, as these were not documented consistently in the database.

Patients were treated with anaesthetic agents at discretion of the emergency physician according to locally established standardized protocols. Exact dosages of anaesthetic substances have not been documented in the electronical database. Both stereoisomers of Ketamine were used by HEMS physicians. However, there was no documentation regarding the discrimination between the S-(+)- and R-(−)-isomer in the database.

Furthermore, two subgroups were defined according to the hypothesis that the choice of an intravenous anaesthetic was influenced by patients suspicious for bleeding trauma or traumatic brain injury. Thus, a subgroup analysis was performed for patients that presented with a Shock Index (SI) >  1 in the pre-hospital assessment. SI was calculated as initial heart rate divided by initial systolic blood pressure [10]. A further subgroup analysis was conducted for patients with a Glasgow Coma Scale (GCS) score ≤ 8 upon initial presentation.

### Database and ethics

For each mission and each patient, information on the pre-hospital course and treatment is documented in an electronical database by the minimal data set for emergency physicians (MIND2). This data set was established by the German Interdisciplinary Association of Intensive Care and Emergency Medicine (DIVI) and contains a basic set of data (characteristics and parameter values). In addition to the MIND2 the ADAC Air Rescue Service collects further data including air-rescue specific parameters.

Study data were extracted in a separate research database (Microsoft®Excel 2010, Microsoft Corporation Redmond, USA). The research database did not provide any personal data (like name, date of birth, etc.). The study was approved by the Central Ethics Commission of the University of Witten/Herdecke (no. 201/2015). Study results are presented according to the STROBE guidelines for observational studies [11].

### Statistics

Formal statistical testing comparing patients within the respective subcohorts was performed using the Chi-Square-Test for trend (SPSS version 18.0 software; SPSS, Chicago, IL, USA). Results here are described as statistically significant if their error probability was less than 5% (*p* < 0.05). Due to the large cohort size even minor differences lead to highly significant results, which could mislead to over-interpretation. The clinical relevance of differences between the observed groups has to be carefully interpreted [12]. All indices are presented as relative referred to the total number of missions for the year, respectively.

## Results section

### Cohort description and administration of anaesthetics

From 2006 until 2015, a total of 11,080 RSI on scene by HEMS physicians after major traumatic injury were documented within the HEMS database (Fig. 1). Cases were excluded if no anaesthetic drug was documented (835 patients) or if more than one intravenous nonopioid anaesthetic was used for induction (525 patients). Thus, between 2006 and 2015, 9720 trauma patients were eligible for the present study.

Overall, the specialty of the emergency physicians displayed following distribution: Anaesthesiology 87%, Surgery 7% and Internal Medicine 5%. The specialty of the emergency physician was not documented for 1%. This distribution did not change over the ten-year study period (data not shown).

According to the on-scene physicians’ assessment, most patients suffered inter alia from life-threatening TBI (34%) as well as in decreasing number of frequency from life-threatening chest (20%), abdominal (11%) or pelvic trauma (7%). In 22%, injuries of the extremities were considered to be life-threatening (Table 1).

**Table 1:** Characteristics of the study population (*n* = 9720)

		Trauma patients (*n* = 9720)
Distribution, *n*		
1-6a		284
7-17a		852
≥ 18a		8558
Age, median (SD) [years]		43 (22)
Male sex, *n* (%*)		7109 (73)
Trauma, life-threatening, *n* (%)*		
TBI		4164 (43)
Chest		1985 (20)
Abdomen		1069 (11)
Pelvis		720 (7)
Extremities		2175 (22)
Vital signs on scene		
Systolic blood pressure, median (SD) [mmHg]		120 (36)
HR, median [bpm] (SD)		100 (25)
Shock Index, *n* (%)	≤ 1	7198 (74)
	> 1	2302 (24)
SpO2	> 90	6997 (72)
	≤ 90	2723 (28)
GCS	> 8	5741 (59)
	≤ 8	3979 (41)

Values are given in absolute numbers (n). In round parentheses relative values referred to the entire cohort (*n* = 9720) or the standard deviation (SD) are presented

**Fig. 2:**
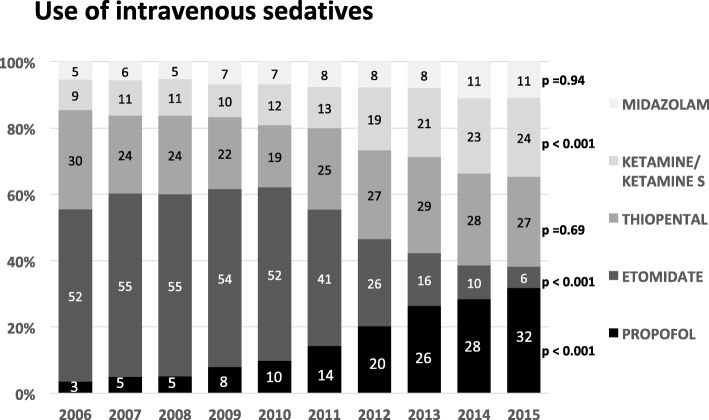
Changes in intravenous sedative use between 2006 and 2015 are presented. For each year the relative distribution of the investigated anaesthetics (Propofol, Etomidate, Thiopental, Ketamin/Ketamin S, Midazolam) referred to the entire cohort (*n* = 9720) is shown

Regardless of the underlying indication, the use of Etomidate decreased dramatically from 52% in 2006 to 6% in 2015 (*p* < 0.001) in favour of a more frequent use of Propofol (3 to 32%, *p* < 0.001) and Ketamine (9 to 24%, *p* < 0.001), particularly since 2010 (Fig. 2). The use of Midazolam increased slightly, while the utilization rate of Thiopental remained constant (30% in 2006, 27% in 2015; *p* = 0.69).

### Administration of anaesthetics in patients with shock index > 1

**Fig. 3:**
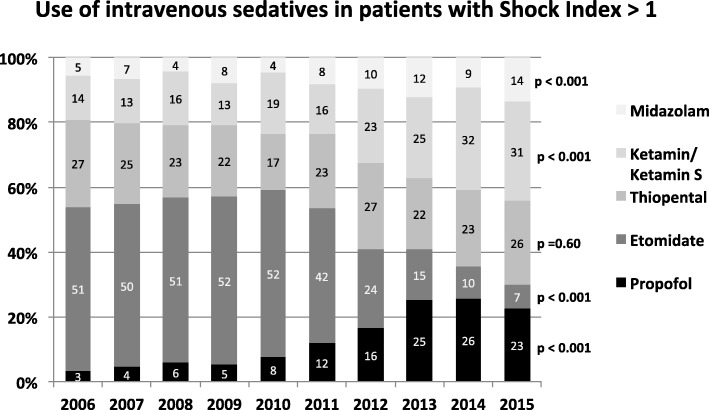
Changes in intravenous sedative use in trauma patients with a Shock Index (SI) > 1 between 2006 and 2015 are presented. For each year the relative distribution of the investigated anaesthetics (Propofol, Etomidate, Thiopental, Ketamin/Ketamin S, Midazolam) referred to the respective cohort (n [SI > 1] = 2302) is shown

Use of sedative agents after trauma was evaluated depending on the circulatory status on scene, as reflected by the Shock Index prior to any treatment (Fig. 3). 24% (*n* = 2302) of patients within our study cohort presented with a SI > 1 following trauma. In patients with SI >  1, the administration rate of Etomidate dropped from 51% in 2006 to 7% in 2015. This decline was mainly substituted by Ketamine and Propofol, while use of Thiopental and Midazolam remained almost constant during the investigation period. Use of Propofol increased especially from 2010 reaching a maximum in 2014 with a rate of 32%.

### Administration of anaesthetics in patients with GCS ≤ 8

**Fig. 4:**
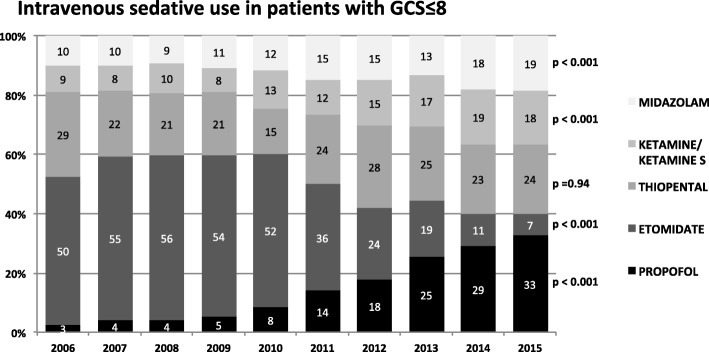
Changes in intravenous sedative use in trauma patients with a GCS ≤ 8 between 2006 and 2015 are presented. For each year the relative distribution of the investigated anaesthetics (Propofol, Etomidate, Thiopental, Ketamin/Ketamin S, Midazolam) referred to the respective cohort (*n* [GCS ≤ 8] = 3979) is shown

Figure 4 summarizes the use of sedative agents in patients suspicious for head injury upon initial contact reflected by a GCS ≤ 8. A similar distribution compared to the general trauma population (Fig. 2) could be observed showing a significant and constant increase of Propofol use for RSI starting in 2009 (Propofol 2009: 5% vs. 2015: 33%, *p* < 0.001). Concordantly, use of Etomidate decreased from 50% in 2006 to 7% in 2015 (Fig. 4). During the investigation period, use of Thiopental exhibited fewer variations showing a relative constant use around 23% (SD 0.04; range between 15 and 29%). For Midazolam and Ketamine, starting in 2009, an ongoing increase could be observed. In contrast to the overall patient population, in patients with GCS ≤ 8 the increase for Ketamine was less pronounced, with a more predominant use of Midazolam.

### Administration of opioids

**Fig. 5:**
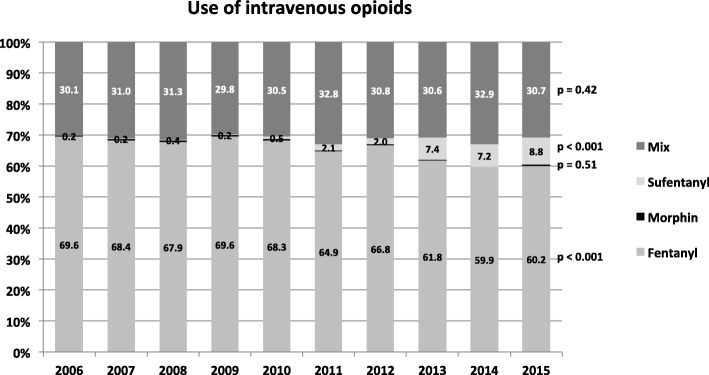
Changes in use of intravenous opioids in the study population (n = 9720) between 2006 and 2015 are presented. For each year the relative distribution of the investigated agent referred to the respective cohort is shown. Morphine was almost not administered. “Mix” means that a mixture of several opioids was administered (e.g. Fentanyl + Morphin)

Figure 5 depicts the administration of opioid agents for the complete study population (*n* = 9720) over the ten-year period, showing that Fentanyl has been the main opioid substance for RSI in trauma patients. However, the use of Fentanyl declined significantly from 2006 (69.6%) to 2015 (60.2%; *p* < 0.001), while administration of Sufentanyl increased significantly (2006: 0.2% vs. 2015: 8.8%, *p* < 0.001). Morphine was almost not used for RSI in trauma patients.

## Discussion

Despite several alternatives of supraglottic airway devices, RSI and intubation of the trachea with a cuffed tube is still recommended for airway management in trauma patients [1]. However, the anaesthetic agent of choice in the prehospital setting remains unclear, also due to differing advantages and disadvantages of each anaesthetic in use. Optimal pharmacokinetic properties for RSI medication include rapid onset, short duration, a minimal side effect profile and, especially for trauma patients, minimal hemodynamic effects [6]. In trauma patients, a secondary aim is to avoid harmful physiological side-effects that may worsen TBI or haemorrhage by episodes of hypotension, hypertension or elevated intracranial pressure [13–15].

To our knowledge, the current study is the first to analyse the usage of anaesthetic agents for trauma patients in a mature physician based EMS system over one decade.

First, the present data from a large pre-hospital database demonstrate that there still appears to be no consensus and a wide variety of on-scene anaesthetics administration.

Secondly, the current study shows a remarkable change from 2006 to 2015, which has only partially been supported by evidence. Particularly, the exponential increase in pre-hospital administration of Propofol and the decrease in inducing patients with Etomidate are obvious. Within our cohort a substantial proportion of patients presented with a shock index > 1 upon primary contact, underlining the relevance of appropriate medication for RSI in trauma. The distribution of the used anaesthetics has changed considerably in this subgroup as well.

Etomidate has been administered frequently, probably as negative effects on circulation are rare. Regarding the changes during the investigation period, particularly since 2011, use of Etomidate decreased substantially by 44%. Actually, the German S3 guideline “Polytrauma” from 2011 advised against the administration of Etomidate due to a potential reversible adrenal insufficiency by dose dependent inhibition of 11ß-hydroxylase [1, 16, 17]. There are still conflicting reports whether a single dose of Etomidate for RSI in trauma causes increased mortality and morbidity at all [18–20]. However, recent studies did not support the German guideline recommendation as a single dose of Etomidate in trauma patients was not found to influence mortality [21–23]. High-level evidence appears necessary to clarify this aspect in trauma [24], because whether suppression of the adrenal axis and increased complication rates seen in several studies result from a common underlying process or whether there is a cause-effect relationship has never been proven.

However, it has to be assumed that the first edition of the German S3 guideline “Polytrauma” from 2011 influenced German emergency physicians decisively, and that there is a strong relationship between these guideline recommendations and the substantial decline in Etomidate use observed in the current study. The guideline generally advises against the use of Etomidate and favours Ketamine for pre-hospital RSI in trauma patients [1]. However, it does not include recommendations on any other anaesthetic agent, irrespective of sedative, opioid or NMBA. For the prehospital setting, Ketamine is recommended as an appropriate anaesthetic for RSI in trauma patients also due to its catecholamine-mediated stabilizing effect on the cardiovascular system [1, 6, 9, 25]. Still, this effect can fail in patients who are catecholamine depleted [6, 26]. Within our cohort, we observed a continuous increase of Ketamine for induction after trauma, which was also apparent within the subgroups (SI > 1 and GCS ≤ 8). This again reflects the German guideline recommendation [1]. Compared to Etomidate, Ketamine appears not to be inferior with respect to providing optimal intubation conditions [20]. Its use in TBI was controversial in the past due to attributed effects on increased intracerebral blood flow, oxygen consumption and intracranial pressure [27, 28]. More recent studies questioned these findings in other fields than RSI [29–31], and additional neuroprotectant capacities due to its ability to antagonize N-methyl-D-aspartate (NMDA) receptors might be beneficial [32]. Nowadays Ketamine is a favourable choice, in compliance with guideline recommendations, even in trauma patients suffering from TBI [1, 6, 9]. With regard to our data, the history of conflicting evidence regarding the effect of Ketamine on intracerebral cellular metabolism might be responsible for a more cautious use on scene in case of a suspected TBI compared to the overall trauma population.

Nowadays, Propofol (2,6 Diisopropylphenol) is by far the most commonly used sedative anaesthetic in the in-hospital setting. In the current study, we observed an impressive increase for Propofol use in pre-hospital trauma care over the ten-year period (2006: 3%, 2015: 32%), which may reflect the existing, large in-hospital experience. However, there is still no evidence that Propofol might be beneficial regarding prehospital anesthesia in trauma patients. Propofol seems to reduce intracranial pressure and raise the convulsive threshold which might be advantageous in several emergency situations. However, a major disadvantage of Propofol is the considerable decrement in MAP during induction by reducing vascular resistance and myocardial negative inotropic effects [6, 33], which might even be detrimental in the hypovolemic trauma patient [33, 34]. In this context, the majority of HEMS physicians (87%) in our study were anaesthesiologists, thus in-hospital practice and experience might have influenced pre-hospital anaesthetic use as well: With the establishment of Propofol as “standard induction sedative” in the in-hospital setting over the last decade, the in-hospital use of barbiturates has fallen dramatically [6]. In contrast to the in-hospital changes, Thiopental is still routinely administered for pre-hospital induction purposes in trauma patients. Thiopental is a favoured anaesthetic for patients suffering from isolated TBI [8], but our data revealed an ongoing broad and constant application of this agent. Presumably, the use of thiopental on scene will drop in the near future as the following generations of HEMS physicians will not able to gain any in-hospital experience with this drug. A similar development could be observed for opioids, as Sufentanyl was almost absent in 2006 and has become increasingly popular over the ten-year period. However, these changes have not been accompanied by any supporting evidence, thus they should be monitored carefully and critically.

In severe TBI, Barbiturates and Propofol are particularly used with regard to reduction in intracranial pressure and cerebral metabolic oxygen consumption rate [6, 8]. On the other hand they have been reported to be associated with poor outcome attributed to a decrease in cerebral metabolism caused by hypotension [35]. Lacking RCTs in the prehospital setting, data from other fields indicate that reduced dosing or administration of pre-procedural volume loading may not be sufficient to maintain adequate mean arterial or cerebral perfusion pressor [34, 36]. In consideration of the above, Propofol and Thiopental have to be used carefully in patients suffering from severe TBI.

Previous studies have not been able to investigate changes in prehospital administration of anaesthetics over a long time period. However, Sunde and colleagues conducted a large study on HEMS in Europe and Australia [37]. This study showed, that the majority of non-cardiac-arrest patients received standard RSI using Opioids, sedatives and NMBA, or an anaesthetic substance plus NMBA. One major difference to our study is that Etomidate is not available in many other countries.

The current study has several limitations. Our data represent a retrospective analysis of a large, preexisting dataset. The listed injury patterns (e.g. TBI, chest) have been documented in the database according to the clinical assessment of the treating emergency physician. Thus, a detailed injury pattern according to the Injury Severity Score cannot be derived with the existing data. Also, the further hospital course or the outcomes are not documented at all. However, previous HEMS studies share the same limitation as well [38], and future investigations will have to examine the effect of the different anaesthetic substances on morbidity and mortality. Furthermore, as a major limitation, use of NMBA was not documented consistently in the database. The dosages of the anaesthetic substances have not been documented in the database, as well as the stereoisomers of Ketamin. Also, we were not able to separate different aetiological entities underlying reduced vigilance after trauma resulting in a GCS ≤ 8. Finally, this study is derived from a large German HEMS database, and cannot be generalized with respect to other European countries. However, anesthetics use in HEMS is presumably representative for German prehospital emergency medicine in general, as medication is not regulated by ADAC or HEMS provider, but depending on respective local policies and preferences of the treating emergency physician.

## Conclusion

This large study analysed intravenous anaesthetics administered for RSI in trauma patients in Germany over one decade, showing a substantial change from 2006 to 2015 despite the lack of any high-level evidence. Etomidate has shifted from the main substance to virtual absence from 2006 until 2015, indicating that the recommendation of an established national guideline was transferred into clinical practice, although based on weak evidence. Thus, the use of other sedatives has increased. Usually applied in the in-hospital setting, the pre-hospital use of Propofol and Sufentanyl showed a particular increase. The induction agent of choice in the pre-hospital setting remains controversial. The mechanisms and advantages of the different substances still have to be elucidated, especially in head injury and bleeding trauma.
